# Copy number alterations in B-cell development genes, drug resistance, and clinical outcome in pediatric B-cell precursor acute lymphoblastic leukemia

**DOI:** 10.1038/s41598-019-41078-4

**Published:** 2019-03-15

**Authors:** Elisabeth M. P. Steeghs, Judith M. Boer, Alex Q. Hoogkamer, Aurélie Boeree, Valerie de Haas, Hester A. de Groot-Kruseman, Martin A. Horstmann, Gabriele Escherich, Rob Pieters, Monique L. den Boer

**Affiliations:** 1 Department of Pediatric Oncology/Hematology, Erasmus Medical Center – Sophia Children’s Hospital, Rotterdam, The Netherlands; 2 Princess Máxima Center for Pediatric Oncology, Utrecht, The Netherlands; 3 DCOG, Dutch Childhood Oncology Group, The Hague, The Netherlands; 4 COALL - German Cooperative Study Group for Childhood Acute Lymphoblastic Leukemia, University Medical Centre Eppendorf, Martinistrasse 52, 20246 Hamburg, Germany

## Abstract

Pediatric B-cell precursor acute lymphoblastic leukemia (BCP-ALL) is associated with a high frequency of copy number alterations (CNAs) in *IKZF1*, *EBF1*, *PAX5*, *CDKN2A/B*, *RB1*, *BTG1*, *ETV6*, and/or the PAR1 region (henceforth: B-cell development genes). We aimed to gain insight in the association between CNAs in these genes, clinical outcome parameters, and cellular drug resistance. 71% of newly diagnosed pediatric BCP-ALL cases harbored one or more CNAs in these B-cell development genes. The distribution and clinical relevance of these CNAs was highly subtype-dependent. In the DCOG-ALL10 cohort, only loss of *IKZF1* associated as single marker with unfavorable outcome parameters and cellular drug resistance. Prednisolone resistance was observed in *IKZF1*-deleted primary high hyperdiploid cells (~1500-fold), while thiopurine resistance was detected in *IKZF1*-deleted primary *BCR-ABL1*-like and non-*BCR-ABL1*-like B-other cells (~2.7-fold). The previously described risk stratification classifiers, i.e. *IKZF1*^plus^ and integrated cytogenetic and CNA classification, both predicted unfavorable outcome in the DCOG-ALL10 cohort, and associated with *ex vivo* drug cellular resistance to thiopurines, or L-asparaginase and thiopurines, respectively. This resistance could be attributed to overrepresentation of *BCR-ABL1*-like cases in these risk groups. Taken together, our data indicate that the prognostic value of CNAs in B-cell development genes is linked to subtype-related drug responses.

## Introduction

Acute lymphoblastic leukemia (ALL) is the most common cancer diagnosed in children. The introduction of risk-adjusted treatment protocols has significantly improved survival rates, which nowadays is approaching 90% survival^1–3^. Outcome of B-cell precursor ALL (BCP-ALL) differs by genetic subtype, i.e. *ETV6*-*RUNX1*, high hyperdiploid, and *TCF3-PBX1* cases have favorable prognosis, whereas *BCR*-*ABL1*, and *KMT2A*-rearranged BCP-ALL is associated with an unfavorable treatment outcome^3^. Approximately 25% of the patients has a genetically unclassified disease, which is defined as ‘B-other’. This heterogeneous group can be subdivided in *BCR-ABL1*-like patients and non-*BCR-ABL1*-like B-other patients^4,5^. Within the *BCR-ABL1*-like subtype intrachromosomal amplification of chromosome 21, dicentric chromosome (9;20), and kinase activating lesions are reported^4–9^. In non-*BCR*-*ABL1*-like B-other cases chromosomal translocations involving *DUX4*, *ZNF384*, and *MEF2D* were identified^10–12^. In addition to the major classifying abnormalities, secondary aberrations have been observed, including copy number alterations (CNAs) in genes involved in B-cell development (e.g. *IKZF1*, *EBF1*, *PAX5*, *ETV6*), cell cycle and proliferation (e.g. *CDKN2A*, *CDKN2B*, *RB1*, *BTG1*), and cytokine receptors (e.g. *CRLF2*)^4,5,8,9,13–16^. Interestingly, some of these genetic lesions (e.g. *IKZF1*) were shown to predict clinical outcome^5,14,17^. The Dutch Childhood Oncology Group (DCOG) implemented *IKZF1* status as risk factor in the ongoing DCOG-ALL11 protocol. In addition, risk stratification strategies were designed by integration of CNA profiles and genetic subtypes^18–20^.

Cellular drug resistance is an important cause of relapse. *Ex vivo* drug resistance at diagnosis is associated with high risk of early treatment failures^21–23^. In addition, BCP-ALL cells at relapse are more resistant towards glucocorticoids, L-asparaginase, anthracyclines, and thiopurines^24^. *IKZF1* deletions are reported to mediate resistance towards glucocorticoids, but the relationship between remaining CNAs and cellular drug resistance is yet unknown^25–27^. Therefore, we performed an explorative study, which aimed to gain insight in associations between CNAs, cellular drug resistance, and clinical outcome.

## Results

**Figure 1:**
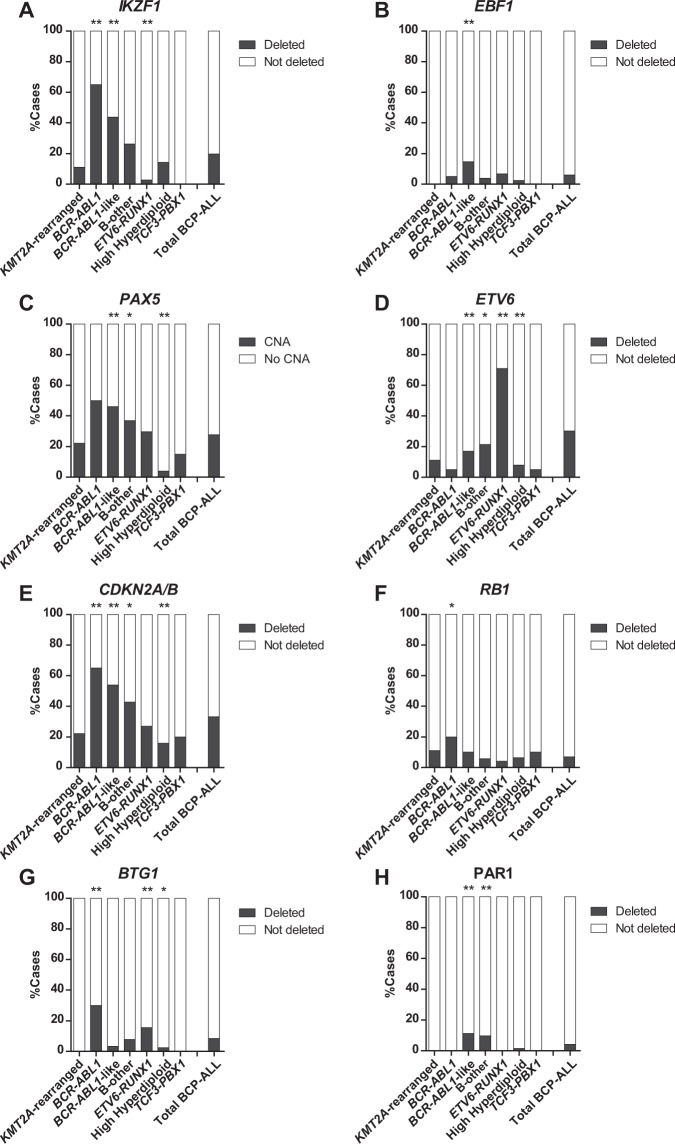
CNA landscape of B-cell development genes in the different subtypes of pediatric BCP-ALL. CNA profile of 515 newly diagnosed pediatric BCP-ALL patients, representing all major BCP-ALL subtypes, was determined using MLPA. Association between CNAs and subtypes was studied using the Fisher Exact test. The proportion of patients per subtype with a specific CNAs is shown. CNAs tested included *IKZF1* (**A**), *EBF1* (**B**), *PAX5* (**C**), *ETV6* (**D**), *CDKN2A/B* (**E**), *RB1* (**F**), *BTG1* (**G**), PAR1 (**H**). **p ≤ 0.01, *p ≤ 0.05. del = deletion.

A pediatric BCP-ALL cohort of 515 newly diagnosed cases, representing all major ALL subtypes, was screened for CNAs in eight genes involved in transcription of lymphoid genes and the differentiation and proliferation of precursor B-cells (henceforth: B-cell development genes; Supplementary Fig. 1). In total, 71% of the pediatric BCP-ALL cases harbored one or more CNAs in these B-cell development genes (Fig. 1). The CNA frequency differed between genetic BCP-ALL subtypes. The percentage of patients with one of more CNAs was the highest in *BCR*-*ABL1*-like cases and the lowest in *TCF3-PBX1* cases (Supplementary Fig. 2A).

### CNAs in B-cell transcription factors

#### IKZF1

**Figure 2:**
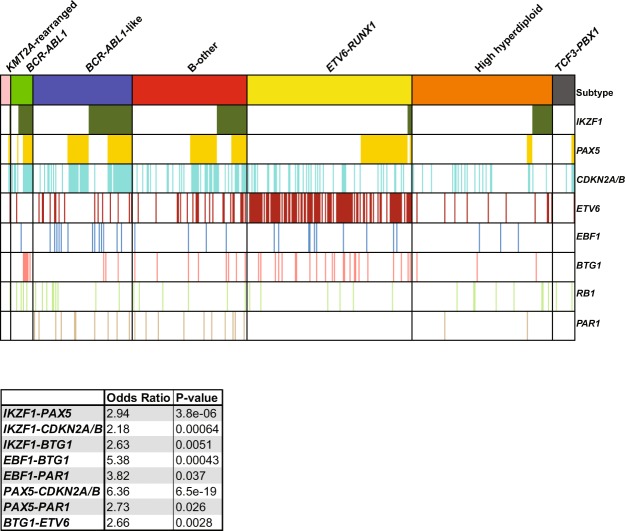
Co-occurence of CNAs in B-cell development genes in the different BCP-ALL subtypes. Heatmap of CNA profile of 515 newly diagnosed pediatric BCP-ALL patients, representing the major BCP-ALL subtypes. CNAs are shown per subtype. Colors indicate presence of a CNA and absence of CNAs is shown in white. The heatmap is sorted on *IKZF1* deletions followed by CNAs in *PAX5*. Each column represents an individual patient. The co-occurrence between the different CNAs in all BCP-ALL cases was calculated using the Fisher Exact test. Odds ratios and p-values of significant associations are shown.

Deletions of the transcription factor *IKZF1* were detected in 20% of the BCP-ALL cases. This frequency differed between subtypes: *IKZF1* deletions were enriched in *BCR-ABL1* (65%) and *BCR*-*ABL1*-like (44%) cases, whereas deletions were low or absent in *ETV6-RUNX1* (3%) and *TCF3-PBX1* (0%), respectively (Fig. 1A; Supplementary Fig. 2B). In addition, 76% (78/102) of the cases with an *IKZF1* deletion harbored CNAs in additional genes, which mainly involved *PAX5* and *CDKN2A/B* (Fig. 2). This co-occurrence was subtype dependent: a strong association (OR >2, p < 0.001) was observed in *BCR*-*ABL1*, *BCR*-*ABL1*-like and B-other cases, whereas in high hyperdiploid cases *IKZF1* deletions mainly occurred independent of CNAs in *PAX5* and/or *CDKN2A/B*. Within the group of genetically unclassified patients, loss of *IKZF1* associated with dicentric chromosome (9;20) and tyrosine kinase fusion genes (Supplementary Table 1).

**Figure 3:**
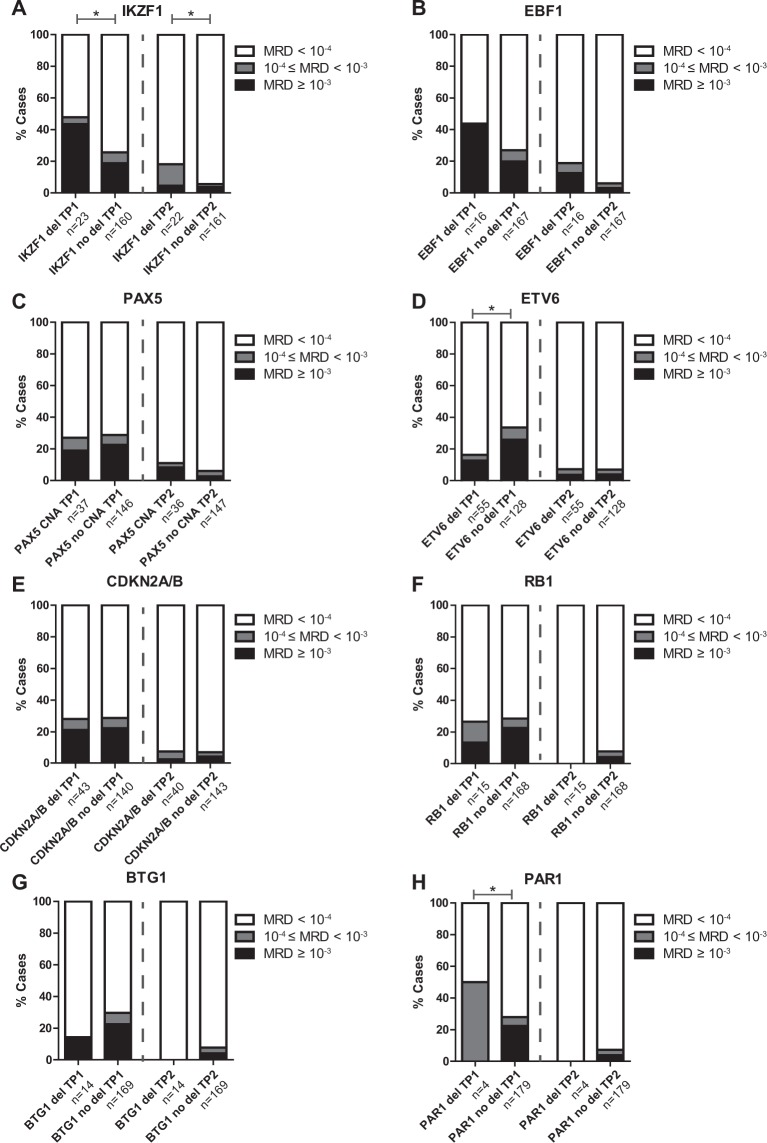
The association between CNAs and MRD levels after induction therapy and the first consolidation course in newly diagnosed BCP-ALL. MRD levels of DCOG-ALL treated BCP-ALL cases (all risk groups) after induction (TP1; n = 183) and first consolidation course (TP2; n = 183). The percentage of cases with high (≥10^−3^), medium (10^−4^ ≤ MRD < 10^−3^), and undetectable MRD levels (<10^−4^) is depicted per CNA. The Fisher’s Exact test was applied to study associations between CNAs and MRD levels. **p ≤ 0.01, *p ≤ 0.05. del = deletion.

**Figure 4:**
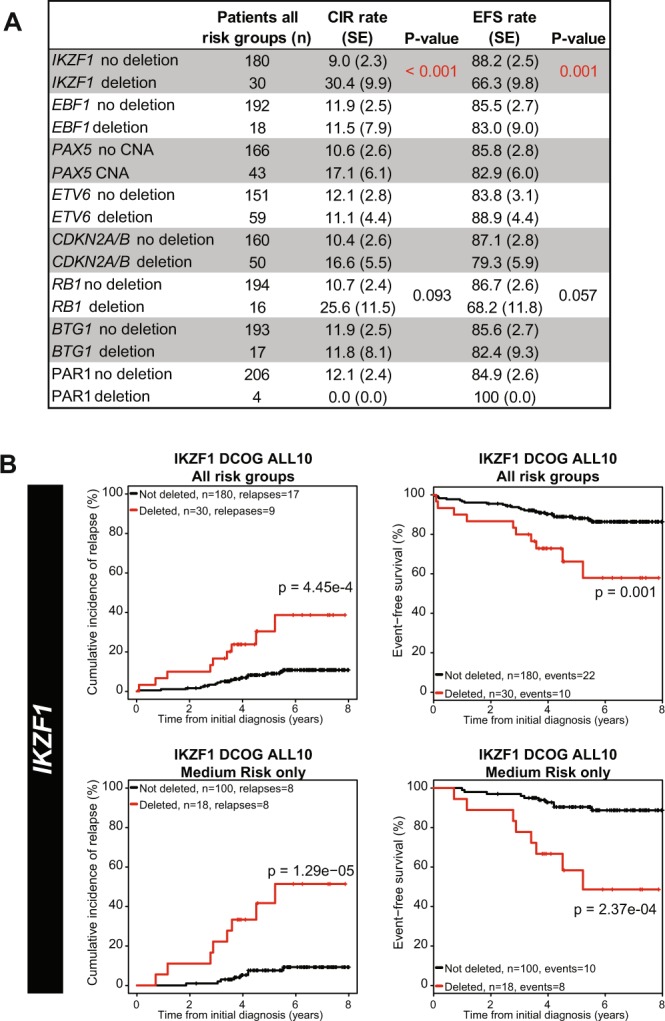
Prognostic value of CNAs in DCOG-ALL10 treated cases. (**A**) The association between CNAs in all risk groups and cumulative incidence of relapse (CIR) and event-free-survival (EFS) was examined. BCP-ALL patients (n = 210) were treated according to DCOG-ALL10 protocol. CIR was estimated using a competing risk model. Relapse and non-response were considered as event, and death as competing event. To test equality of the CIRs, the Gray’s test was applied. Non-response, relapse, and death were considered as events for EFS. EFS rates were determined using Cox regression, and compared using the Wald test. For reliable test results, groups should contain at least 5 cases. (**B**) CIR and EFS curves of cases without or with an *IKZF1* deletion. Curves contain either all risk groups, or the medium risk arm only.

*IKZF1*-deleted cases more often showed high MRD levels (≥10^−3^) after induction therapy (TP1; p = 0.013), and intermediate MRD levels (10^−4^ ≤ MRD < 10^−3^) after the first consolidation course (TP2; p = 0.028), compared to *IKZF1*-wildtype cases (Fig. 3A). This association could be predominantly attributed to high MRD levels in *BCR-ABL1*-like and B-other cases, but was not observed in high hyperdiploid or *ETV6-RUNX1* cases (Supplementary Figs 3–6). In addition, *IKZF1*-deleted cases more often suffered from a non-response or relapse compared to *IKZF1*-wildtype cases (5-year CIR: 30.4% versus 9.0%; p < 0.001; Fig. 4A,B), confirming previous findings^5,14,17^. An *IKZF1* deletion remained predictive for an unfavorable outcome in DCOG-ALL10 cases treated in the medium risk arm (Fig. 4B), indicating that the prognostic value of *IKZF1* is independent of the early treatment response monitored by MRD.

**Figure 5:**
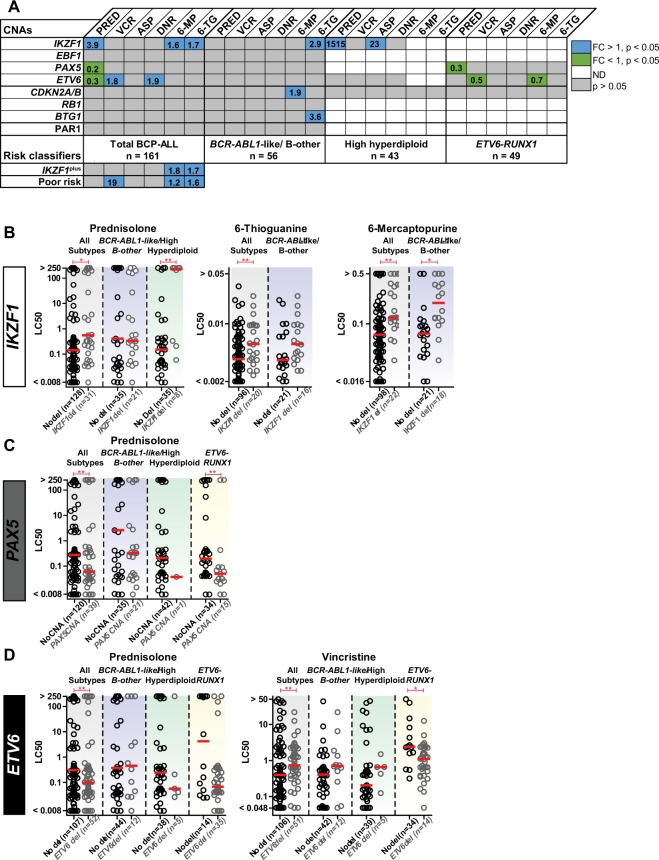
The association between CNAs and the *ex vivo* cellular drug response. (**A**) Leukemic cells were incubated for four days with a concentration range of prednisolone (µg/ml), vincristine (µg/ml), L-asparaginase (IU/ml), daunorubicin (µg/ml), 6-mercaptopurine (µg/ml), and 6-thioguanine (µg/ml), after which cell viability was measured using an MTT assay. The Mann-Whitney U test was applied to compare LC50-values. No association is depicted in grey, resistance in blue (p < 0.05, fold induction (FI) > 1), sensitive in green (p < 0.05, FI < 1), and not determined in white. The number of cases that were tested for prednisolone is depicted, and represent the maximum number of cases. For reliable test results, groups should contain at least 5 cases (groups ≤ 5 are depiced as ND). Results of single CNAs are depicted for all risk groups and for *BCR-ABL1*-like/B-other cells, high hyperdiploid cells, and *ETV6-RUNX1* cells. In addition, associations between the risk classifiers *IKZF1*^plus^ and integrated cytogenetic and CNA classification (poor risk) and cellular drug resistance are shown^18,20^. (**B**) LC50 values for prednisolone (µg/ml), 6-thiogunanine (µg/ml), and 6-mercaptopurine (µg/ml) of cases without or with *IKZF1* deletion. Columns include all BCP-ALL subtypes (grey), *BCR-ABL1*-like/B-other cells (blue), and high hyperdiploid cells (green). The red line represent the median LC50 value in the each group. (**C**) LC50 values for prednisolone (µg/ml) of cases without or with *PAX5* CNAs. Columns include all BCP-ALL subtypes (grey), *BCR-ABL1*-like/B-other cells (blue), high hyperdiploid cells (green), and *ETV6-RUNX1* cells (yellow). The red line represent the median LC50 value in the each group. (**D**) LC50 values for prednisolone (µg/ml), vincristine (µg/ml), and daunorubicin (µg/ml) of primary leukemic cells without or with *ETV6* deletion. Columns include all BCP-ALL subtypes (grey), *BCR-ABL1*-like/B-other cells (blue), high hyperdiploid cells (green), and *ETV6-RUNX1* cells (yellow). The red line represent the median LC50 value in the each group. *p < 0.05, **p < 0.01; Mann-Whitney U test.

As cellular drug resistance might underlie this poor outcome, we examined the *ex vivo* efficacy of chemotherapeutic agents that are commonly used during induction and consolidation therapy. Primary BCP-ALL cells harboring *IKZF1* deletions were more resistant to prednisolone and thiopurines compared to *IKZF1* wildtype cells (p < 0.05; Fig. 5A,B). Resistance against these agents was subtype dependent, as visualized in Fig. 5B: prednisolone resistance was predominantly observed in high hyperdiploid cells (~1500-fold, p = 0.009), whereas thiopurine resistance (6-thioguanine (1.6 fold, p = 0.011) and 6-mercaptopurine (1.7 fold, p < 0.001)) was mainly identified in *IKZF1*-deleted *BCR-ABL1*-like and B-other cells (Fig. 5B). Moreover, high hyperdiploid cells with a deletion of *IKZF1* were more resistant to L-asparaginase(Supplementary Fig. 8A).

#### EBF1

The transcription factor *EBF1* was deleted in a minority (6%) of the BCP-ALL cases (Fig. 1B). Deletions were enriched in *BCR-ABL1*-like cases (15%), but absent in *KMT2A*-rearranged and *TCF3-PBX1* cases (Fig. 1B). Cases that harbored an *EBF1* deletion showed a trend towards higher MRD levels after induction therapy compared to non-*EBF1* deleted cases (OR = 5.16, p = 0.057; Fig. 3B). No association between loss of *EBF1* and cellular drug resistance was observed, though drug resistance data were only available for six *EBF1* deleted cases.

#### PAX5

CNAs of the transcription factor *PAX5* were observed in 28% of the BCP-ALL cases (Fig. 1C). CNAs were detected throughout all BCP-ALL subtypes, although the frequency was relatively high in *BCR-ABL1* (50%) and *BCR-ABL1*-like (46%) cases (Fig. 1C). The strong co-occurrence of *PAX5* and *CDKN2A/B* CNAs (Fig. 2, OR = 6.36, p < 0.001) is likely caused by the high frequency of chromosome 9p deletions observed in these cases^28^; the chromosome arm on which PAX5 and CDKN2A/B are located. In correspondence, chromosome 9p deletions were observed in 51.8% (44/85) of these cases. Despite of the strong association between *PAX5* CNAs and *IKZF1* deletions (Fig. 2, OR = 2.94, p < 0.001), CNAs in *PAX5* were not predictive for high MRD levels (Fig. 3C) nor a poor prognosis in pediatric BCP-ALL cases (Fig. 4A). Strikingly, leukemic cells harboring CNAs in *PAX5* showed an increased sensitivity (~5.1 fold, p = 0.008) to prednisolone compared to *PAX5*-wildtype cells (Fig. 5C). This difference in sensitivity was only significant (p = 0.031) in *ETV6-RUNX1* cases, but a similar pattern was also observed in the remaining BCP-ALL subtypes (Fig. 5C). Interestingly, this association depended on the *IKZF1* status: cells with both an *IKZF1* deletion and a *CNA* in *PAX5* were equally sensitive to prednisolone as *IKZF1* and *PAX5* wildtype cells, whereas cells with only an *IKZF1* deletion were more resistant to prednisolone (Supplementary Fig. 8B). These results suggest that CNAs in *PAX5* might compensate for prednisolone resistance induced by loss of *IKZF1*.

#### ETV6

Deletions of the transcription factor *ETV6* were detected in all BCP-ALL subtypes, but were especially enriched in *ETV6-RUNX1* cases (71%; Fig. 1D). After induction therapy (TP1), *ETV6*-deleted cases more often showed low (<10^−4^) MRD levels compared to *ETV6-*wildtype cases (Fig. 3D; OR = 2.6, p = 0.02). However, this association was subtype dependent: in *BCR-ABL1*-like and B-other cases an adverse association between *ETV6* deletions and MRD levels was observed (Supplementary Fig. 3). Prognosis of cases with loss of *ETV6* was not different compared to *ETV6*-wildtype cases (Fig. 4). *ETV6*-deleted cells appeared to be more sensitive to prednisolone (~3.2 fold, p = 0.046), but more resistant to vincristine (~1.8 fold, p < 0.01) and daunorubicin (~1.9 fold, p = 0.028). Remarkably, loss of the wildtype *ETV6* allele in *ETV6*-*RUNX1*-positve cells associated with a high sensitivity to vincristine instead of resistance (p = 0.013, Fig. 5D), suggesting that associations of vincristine resistance differ between genetic subtypes of ALL. Moreover, deletion of *ETV6* was associated with L-asparaginase resistance in high hyperdiploid cells and high 6-thioguanine sensitivity in *ETV6*-*RUNX1* cells (Supplementary Fig. 8C).

### CNAs in cell cycle and proliferation genes

#### CDKN2A/B

Deletions of the cell cycle regulators *CDKN2A* and/or *CDKN2B* were often observed (33%) in the pediatric BCP-ALL cohort (Fig. 1E). Similar to *PAX5*, the deletions in *CDKN2A/B* were found in all BCP-ALL subtypes, but were especially enriched in *BCR-ABL1* (65%, OR = 3.95, p = 0.003), *BCR-ABL1*-like (54%, OR = 2.88, p < 0.001), and B-other cases (OR = 1.67, p = 0.026). No association with clinical outcome parameters or cellular drug resistance was observed (Figs 3–5).

#### RB1

The cell cycle regulator *RB1* was deleted in a minority (~7%) of the BCP-ALL cases (Fig. 1F) and deletions were detected in all BCP-ALL subtypes. Within the DCOG-ALL10 cohort, *RB1*-deleted cases showed a trend towards a poor event free survival (5- year EFS: 68.2% versus 86.7%, p = 0.057), which was caused by an unfavorable response in the medium risk (MR) treatment group (5-year EFS: 46.9% versus 88.3%, p = 0.003; Supplementary Fig. 7A). No association with MRD levels or cellular resistance to the tested drug panel was observed (Fig. 5).

#### BTG1

The anti-proliferative gene *BTG1* was deleted in a minority (~8%) of the BCP-ALL cases. No deletions were detected in *KMT2A*-rearranged or *TCF3-PBX1* cases, whereas the highest frequency was observed in *BCR-ABL1* (30%) and *ETV6-RUNX1* (16%) cases (Fig. 1G). Four out of five *BTG1*-deleted *BCR*-*ABL1*-like and B-other cases also harbored an *IKZF1* deletion. These four cases all experienced an event and only the patient with wildtype *IKZF1* remained in remission (Supplementary Fig. 7B). This finding underlines an earlier report, in which a cooperative effect of *BTG1* and *IKZF1* lesions in leukemogenesis was observed^27^.

### CNAs in cytokine receptors

#### PAR1

Deletions in the pseudoautosomal region 1 (PAR1) were the least prevalent (~4%) in this pediatric BCP-ALL cohort. CNAs in this region indicate presence of interstitial deletions or a translocation, which both induce overexpression of *CRLF2*^29^. Deletions of the PAR1 region were detected in *BCR*-*ABL1*-like (11%), B-other (10%), and high hyperdiploid cases (2%), but not in remaining BCP-ALL subtypes (Fig. 1H). Unfortunately, power was lacking to reliable study the association between deletions in the PAR1 region, MRD levels, clinical prognosis, and cellular drug resistance.

Taken together, with the exception of loss of the *IKZF1* gene, none of the CNAs in the remaining B-cell development genes strongly associates with clinical outcome and cellular drug resistance as single marker. Our results show that the clinical value of CNAs in B-cell development genes is highly context dependent and differs between the diverse oncogenic drivers of pediatric BCP-ALL.

### Risk stratification classifiers

In recent studies, *IKZF1*^plus^ and integrated cytogenetic and CNA classification were shown to be prognostic classifiers^18,20^. In the DCOG-ALL10 cohort 12 of the 210 cases were classified as *IKZF1*^plus^^20^. The prognosis of *IKZF1*^plus^ cases was unfavorable compared to cases with wildtype *IKZF1* (Supplementary Fig. 9). Strikingly, no prednisolone resistance was observed in *IKZF1*^plus^ cells, which could be explained by underrepresentation of high hyperdiploid cases in this group (n = 1, Fig. 5A). However, *IKZF1*^plus^ cases did show *ex vivo* resistance to 6-thioguanine and 6-mercaptopurine, mainly caused by the high proportion of *BCR*-*ABL1*-like and B-other cases in this group.

Integration of cytogenetic and CNA data as reported by Moorman *et al*.^18^ identified cases with a genetic good and poor risk. Cases that were classified as poor risk showed an unfavorable 5-years EFS and CIR compared good risk cases, as shown in Supplementary Fig. 10A. These genetically poor risk cases showed high MRD levels after induction therapy and the first block of consolidation therapy, indicating a poor response to drugs that are used during these treatment phases (Supplementary Fig. 10B). Indeed, *ex vivo* cellular drug response data showed resistance of poor risk cells to L-asparaginase, 6-thioguanine, and 6-mercaptopurine (Fig. 5A, Supplementary Fig. 10C). Enrichment of *BCR*-*ABL1*-like cases could attribute to the thiopurine and L-asparaginase resistance observed in the poor risk group^4^.

## Discussion

BCP-ALL cases harbor various genetic aberrations in genes involved in lymphoid maturation, cell cycle regulators, tumor suppressors, and tyrosine kinases. We performed an explorative study to gain insight in the association between CNAs in B-cell development genes, MRD levels, long-term prognosis, and cellular drug resistance. Interestingly, the distribution and clinical relevance of these CNAs was subtype-dependent. A high frequency of CNAs in these B-cell development genes was found in the poor prognostic subtypes *BCR-ABL1*, *BCR*-*ABL1*-like, and B-other. Cooperative lesions may favor the aggressive phenotype of a leukemia, such as exemplified by the synergistic effect between loss of *IKZF1* and the *BCR*-*ABL1* fusion gene in leukemogenesis^30^, and the antagonizing effect of *IKZF1* deletions in the response to imatinib^31^. In contrast, the prognosis of *ETV6*-*RUNX1*, *DUX4*-rearranged, and *ERG*-deleted BCP-ALL is probably not affected by *IKZF1* deletions, but numbers with *IKZF1* deletions in these subtypes are low^11,12,15,32,33^. These observations indicate that the genetic context influences the functional effect of CNAs in B-cell development genes. The importance of the genetic context is exemplified by the fact that isolated deletions of *BTG1* do not affect cellular drug resistance or the prognosis of BCP-ALL cases, whereas all four patients with concomitant loss of *BTG1* and *IKZF1* experienced an event. Moreover, combined loss *BTG1* and *IKZF1* was shown to enhance glucocorticoid resistance^27^. In contrast to *BTG1-IKZF1* synergy, we observed that CNAs in *PAX5* may counteract the effect of an *IKZF1* deletion on prednisolone resistance. Various combinations of cooperative lesions may therefore have different effects on the pathobiology of B-cell precursor ALL cells.

In the present study we observed an association between deletion of *IKZF1* and prednisolone resistance, especially in high hyperdiploid cells. In correspondence, a direct association has been demonstrated between *IKZF1* deletion and glucocorticoid-induced cell death^25,34^. *IKZF1* functions as a metabolic gatekeeper and consequently loss of *IKZF1* results in increased intracellular ATP and glucose levels^34^. Interestingly, we previously observed a direct relation between an increased glycolytic rate and prednisolone resistance in primary BCP-ALL cells^35,36^. In these leukemic cells, inhibition of glycolysis restored the efficacy of prednisolone^36^. Hence, inhibition of glycolysis might also be a potential treatment strategy to re-sensitize *IKZF1*-deleted cells to prednisolone and should be explored in more detail in future studies, also in the context of *BTG1* and *PAX5*.

In contrast to high hyperdiploid cells, deletion of *IKZF1* was not linked to prednisolone resistance in primary *BCR-ABL1*-like and B-other ALL cells, suggesting that additional factors (e.g. differentiation stage, other oncogenic drivers) are important for the functional effect of a deletion of the IKZF1 gene in these type of cells. Instead of prednisolone resistance, we observed thiopurines resistance in these *BCR-ABL1*-like and B-other ALL cells. Thiopurine resistance might be caused by deficiencies in the DNA mismatch repair system and indeed DNA repair genes were reported to be downregulated in *IKZF1*-deleted cells^37,38^. Interestingly, this characteristic might offer opportunities to target these leukemic cells via the DNA mismatch repair apparatus, e.g. by PARP inhibitors, and warrants further studies.

Besides *IKZF1*, deletion of *RB1* was predictive for a poor outcome in the MR-risk group of the DCOG-ALL10 cohort. *RB1* deletions are known to be enriched in poor prognostic iAMP21 and hypodiploid cases, which might explain the unfavorable outcome of RB1-deleted cases^13,39,40^. However, the unfavorable outcome could not be explained by or cellular resistance against induction therapy drugs.

Recently, two independent studies showed that integration of genetic aberrations improved the risk stratification of BCP-ALL in children^18,20^. Both *IKZF1*^plus^ and integrated cytogenetic and CNA classification predicted poor outcome in the DCOG-ALL10 cohort, and associated with drug resistance to thiopurines, or L-asparaginase and thiopurines, respectively. The cellular drug resistance could be attributed to overrepresentation of *BCR*-*ABL1*-like cases in these risk groups^4^. Taken together, our results suggest that the prognostic value of CNAs in B-cell development genes is linked to subtype-related drug resistance.

In the current study, we restricted our analyses to CNAs in eight genes that are recurrently deleted in pediatric BCP-ALL. However, additional genetic aberrations may be of importance for prognosis and cellular drug resistance and should be explored in future research. Moreover, as we performed an explorative study, it is of importance to confirm the associations that are proposed in the present paper in independent studies.

In conclusion, results obtained in the present study revealed that, with the exception of an *IKZF1* deletion, none of the remaining CNAs as single marker associated both with an unfavorable clinical prognosis and cellular drug resistance. Our results indicate that the biological and clinical importance of CNAs in B-cell development genes (and presumably also other genetic aberrations) is highly context dependent and differs between the diverse oncogenic drivers of pediatric BCP-ALL. Functional studies that focus on potential causes of cellular drug resistance should therefore take the oncogenic driver and additional genetic aberrations into account.

## Methods

### Processing of primary patient material

Bone marrow and/or peripheral blood samples were obtained from children (1–18 years) with newly diagnosed ALL. Written informed consent was obtained from parents or guardians to use excess of diagnostic material for research purposes, as approved by the Medical Ethics Committee of the Erasmus Medical Center, The Netherlands. These studies were conducted in accordance with the Declaration of Helsinki. Mononuclear cells were isolated using Lymphoprep gradient separation and the leukemic blast percentage was determined microscopically by May-Grünwald Giemsa stained cytospin preparations, as described previously^21^. Samples were enriched to over 90% purity of leukemic cells by depletion of non-leukemic cells using immunomagnetic beads. Primary leukemic cells were maintained in RPMI-1640 Dutch modification supplemented with 20% fetal calf serum (Integro), with 0.1% insulin-transferrin-sodium selenite (Sigma), 0.4 mM glutamine (Invitrogen), 0.25 μg/ml gentamycine (Gibco), 100 IU/ml penicillin (Gibco), 100 μg/ml streptomycin (Gibco), 0.125 μg/ml fungizone (Gibco).

The major cytogenetic subtypes, i.e. high hyperdiploid (>50 chromosomes), *ETV6-RUNX1*, *TCF3-PBX1*, *KMT2A*-rearranged, *BCR-ABL1*, *BCR-ABL1*-like, and B-other (negative for all before mentioned genomic lesions), were determined using fluorescent *in situ* hybridization and (RT-)PCR. The 110-probeset gene expression classifier was used to identify *BCR-ABL1*-like cases^4^. Patients were treated according to the Dutch Childhood Oncology Group (DCOG)-ALL8, -ALL9, -ALL10, the EsPhALL protocol, or the COALL-06-97 and COALL-07-03 study protocols^3,14,41,42^. Patient characteristics were provided by the central study centers of DCOG, The Hague, the Netherlands and COALL, Hamburg, Germany. PCR-detected MRD was evaluated according to the EuroMRD guidelines^3,43,44^.

### Multiplex Ligation-Dependent Probe Amplification

To identify genomic lesions in *IKZF1*, *CDKN2A*, *CDKN2B*, *ETV6*, *PAX5*, *RB1*, *BTG1*, *EBF1,* and the PAR1 region (*CSF2RA/IL3RA/CRLF2*), the SALSA P335 ALL-*IKZF1* (a3) and the SALSA P202 Multiplex Ligation-dependent Probe Amplification (MLPA) assays (MRC-Holland, Amsterdam, Netherlands) were used as described previously^14^. In short, DNA fragments with incorporated FAM nucleotides were generated using 125 ng of genomic DNA, according to the manufacturer’s protocol. To quantify the amplified fragments, an ABI-3130 genetic analyzer (Applied Biosystems, Carlsbad, CA) was used. The manufacturer’s control probes were used to normalize peak intensities, as well as a synthetic control reference generated from five normal DNA samples in the same MLPA run (normal copy number = 0.75 ≤ peak ratio ≤ 2.0; deletions = peak ratio < 0.75; gain = peak ratio > 2.0). A deletion was defined by a peak ratio below 0.75 for at least one MLPA-probe per gene. CDKN2A/B deletions included loss of either *CDKN2A* or *CDKN2B*. The effect of intragenic amplifications and/or deletions in *PAX5* were analyzed within one group, as they were predicted to be functionally equivalent^18,45^. Loss of the PAR1 region was defined by deletion of both *IL3RA* and *CSF2RA* probes while expression of the *CRLF2* and *SHOX*-*AREA* probes was maintained. MLPA analyses were performed in 515 BCP-ALL cases, representing the major genetic subtypes in childhood ALL, i.e. 3.9% *BCR*-*ABL1*, 17.3% *BCR-ABL1*-like, 20.2% non-*BCR*-*ABL1*-like B-other, 28.7% *ETV6*-*RUNX1*, 24.5% high hyperdiploid, 1.6% *KMT2A-*rearranged, 3.9% *TCF3-PBX1*.

### Clinical characteristics and statistics

To identify whether CNAs were underrepresented or enriched in a subtype, the Fisher’s exact test was applied using R software (version 3.2.1). Obtained odds ratios (ORs), 95% confidence interval, and p-values are reported. The Fisher’s exact test was also applied to compare minimal residual disease (MRD) levels after induction and first consolidation therapy between patients groups with CNAs and wildtype patients. Cumulative incidence of relapse (CIR) was estimated using a competing risk model and significance was determined using the Gray’s test. Relapse and non-response (counted as event at day 79) were considered as event, with death as competing event. Event-free survival (EFS) probabilities were estimated using cox regression and compared using the Wald test. Relapse, non-response, secondary malignancies and death were counted as events. Outcome analyses were performed in R (version 3.2.1), using the packages cmprsk version 2.2–7^46^, mstate version 0.2.7^47^ and survival version 2.38–4^48^. Five-year EFS and CIR are reported. The DCOG-ALL10 trial is the most recently completed nationwide trial in which patients were risk-stratified by minimal residual disease (MRD) levels and for whom sufficient long-term follow-up data were available. Therefore, we restricted the analysis of associations between CNAs and clinical response parameters (MRD, clinical outcome) to this cohort. In addition, the genetic subtypes are represented with a distribution that is comparable to the general pediatric BCP-ALL population (excluding *BCR-ABL1*-positive cases since these patients are eligible for the EsPhALL protocol), i.e.12.2% *BCR-ABL1*-like, 13.9% non-*BCR*-*ABL1*-like B-other, 33.5% *ETV6-RUNX1*, 32.7% high hyperdiploid, 2.0% *KMT2A-*rearranged, and 5.7% *TCF3-PBX1* positive cases. The clinical characteristics of this cohort are displayed in Supplementary Table 2.

### *Ex vivo* drug resistance assays

*Ex vivo* cytotoxicity of prednisolone, vincristine, L-asparaginase, daunorubicin, 6-mercaptopurine, and 6-thioguanine was evaluated using 3-(4,5-dimethylthiazolyl-2)-2,5-diphenyltetrazolium bromide (MTT), as previously described^21^. In brief, cells were exposed to a concentration range of chemotherapeutics (prednisolone: 0.008 to 250 μg/mL; vincristine: 0.05 to 50 μg/mL; L-asparaginase: 0.003 to 10 IU/mL; daunorubicin: 0.002 to 2 μg/ml; 6-mercaptopurine: 15.6 to 500 μg/ml; and 6-thioguanine: 1.56 to 500 μg/ml) in a 96 wells plates for four days at 37 °C and 5% CO_2_. After four days of culture, samples were included if control wells harbored more than 70% leukemic cells and an optical density higher than 0.050 arbitrary units (adjusted for blank values). The concentration of drug lethal to 50% of the cells (LC50) was calculated. LC50-values were compared by the Mann-Whitney U test and adjusted for tied ranks if applicable.

## Supplementary information

Supplementary Information

## Data Availability

The datasets generated during and/or analyzed during the current study are available from the corresponding author on reasonable request.
