# Supplementary material for: Copy number alterations in B-cell development genes, drug resistance, and clinical outcome in pediatric B-cell precursor acute lymphoblastic leukemia
Source: Sci Rep. 2019 Mar 15;9:4634. doi: 10.1038/s41598-019-41078-4 (PMC6420659; doi:10.1038/s41598-019-41078-4)

Supplemental Data

Copy number alterations in B-cell development genes, drug resistance, and clinical outcome in pediatric B-cell precursor acute lymphoblastic leukemia

Elisabeth M.P. Steeghs[1], Judith M. Boer[1,2], Alex Q. Hoogkamer[1], Aurélie Boeree[1], Valerie de Haas[3], Hester A. de Groot-Kruseman[3], Martin A. Horstmann[4], Gabriele Escherich[4], Rob Pieters[2,3], and Monique L. den Boer[1,2,3,*]

[1] Department of Pediatric Oncology/Hematology, Erasmus Medical Center – Sophia Children's Hospital, Rotterdam, the Netherlands

[2] Princess Máxima Center for Pediatric Oncology, Utrecht, The Netherlands.

[3] DCOG, Dutch Childhood Oncology Group, The Hague, The Netherlands

[4] COALL - German Cooperative Study Group for Childhood Acute Lymphoblastic Leukemia, University Medical Centre Eppendorf, Martinistrasse 52, 20246 Hamburg, Germany

Running short title: CNAs in B-cell development genes in pediatric ALL

Correspondence:

Monique L. den Boer, PhD

Princess Máxima Center for Pediatric Oncology

P.O. Box 113

NL-3720 AC Bilthoven

Phone: +31 88 97 272 72

Supplementary Table 1: Genomic lesions in *BCR-ABL1*-like and B-other cases

	BCR-ABL1-like			B-other			*BCR-ABL1*-like and B-other		
	IKZF1 del	No *IKZF1* del	p-value	*IKZF1* del	No *IKZF1* del	p-value	*IKZF1* del	No *IKZF1* del	p-value
**Tyrosine kinase fusion**	24.2% (8/33)	12.2% (5/41)	0.23	0% (0/13)	0% (0/58)	1	17.4% (8/46)	5.1% (5/99)	**0.026**
***ERG* deletion**	0% (0/31)	0% (0/41)	1	4.2% (1/24)	14.0% (8/57)	0.268	1.8% (1/55)	8.1% (8/99)	0.16
**Dic(9;20)**	34.2% (12/35)	14.6% (7/48)	0.06	3.8% (1/26)	2.9% (2/68)	1	21.3% (13/61)	7.8% (9/116)	**0.015**
**iAMP21**	8.6% (3/35)	18.8% (9/48)	0.23	3.8% (1/26)	1.5% (1/68)	0.479	6.6% (4/61)	8.6% (10/116)	0.77
**Chromosome 9p deletion**	40% (14/35)	25% (12/48)	0.16	19.2% (5/26)	16.2% (11/68)	0.763	31.1% (19/61)	19.8% (23/116)	0.10

Supplementary Table 2: Clinical characteristics of the DCOG-ALL10 cohort

**Clinical Features**		**DCOG-ALL10 n=210**
**Male**		108/210 (51%)
**age ≥ 10 years**		37/210 (18%)
**WBC ≥ $50x10^9$/L**		25/207 (12%)
**Risk arm MR or HR**		139/201 (69%)
**Prednisone poor response[a]**		11/203 (5%)
**No CR after induction[b]**		2/199 (1%)
**NCI high risk[c]**		65/209 (31%)
**MRD TP1[d]**	high	39/181 (22%)
	intermediate	12/181 (7%)
	low	130/181 (72%)
**MRD TP2[d]**	high	7/181 (4%)
	intermediate	6/181 (3%)
	low	168/181 (93%)

[a] Prednisone response on day 8 ≥$1.0x10^9$ blasts/L (DCOG ALL-10 protocols).
[b] Complete remission (CR) after induction (day 33 in DCOG ALL-10) is defined on morphological grounds by the presence of <5% leukemic blasts and regenerating hematopoiesis.
[c] NCI-Rome high risk is defined by white blood cell count (WBC) ≥_$50x10^9$/L and/or age ≥ 10 years.
[d] Minimal residual disease PCR high (≥10^{-3}), intermediate (≥10^{-4} and <10^{-3}), low (<10^{-4}); TP1, after the first induction course of chemotherapy, day 33; TP2, before consolidation, day 79.

Supplementary Figure Legends

Supplementary Figure 1. Overview of cohort screened for CNAs.

Flowchart showing the number of cases tested for CNAs in *IKZF1*, *EBF1*, *PAX5*, *ETV6*, *CDKN2A/B*, *RB1*, *BTG1*, and the PAR1 region. Cells from a selection of cases were used to study the drug sensitivity. The prognostic relevance of the CNAs (i.e. long-term prognosis and MRD levels) were studied in DCOG-ALL10 cases.

Supplementary Figure 2. CNA landscape of B-cell development genes in the different subtypes of pediatric BCP-ALL.

CNA profile of 515 newly diagnosed pediatric BCP-ALL patients, representing the major BCP-ALL subtypes, was determined using MLPA.

(**A**) The frequency of any CNA (*IKZF1*, *EBF1*, *PAX5*, *CDKN2A/B*, *RB1*, *BTG1*, *ETV6*, and/or PAR1) is shown per subtype. One CNA was sufficient to be quantified as altered. **p≤0.01

(**B**) The association between *IKZF1* deletions and BCP-ALL subtypes. *IKZF1* deletions are depicted as deletion of exon 4-7, deletions of exon 1-8, or remaining deletions.

Supplementary Figure 3. The association between CNAs and MRD levels after induction therapy and the first consolidation course in *BRC-ABL1*-like and B-other patients.

MRD levels of DCOG-ALL treated *BCR-ABL1*-like and non-*BCR-ABL1*-like B-other cases after induction (TP1; n=47) and first consolidation course (TP2; n=46). The percentage of cases with high ($\geq 10^{-3}$), medium (10^{-3} > MRD ≥ 10^{-4}), and undetectable MRD levels ($<10^{-4}$) is depicted per CNA.

Fisher's Exact test. **p≤0.01, *p≤0.05. del = deletion.

Supplementary Figure 4. The association between CNAs and MRD levels after induction therapy and the first consolidation course in high hyperdiploid patients.

MRD levels of DCOG-ALL treated high hyperdiploid cases after induction (TP1; n=60) and first consolidation course (TP2; n=59). The percentage of cases with high ($\geq 10^{-3}$), medium (10^{-3} > MRD ≥ 10^{-4}), and undetectable MRD levels ($<10^{-4}$) is depicted per CNA.

Fisher's Exact test. **p≤0.01, *p≤0.05. del = deletion.

Supplementary Figure 5. The association between CNAs and MRD levels after induction therapy and the first consolidation course in *ETV6-RUNX1* patients.

MRD levels of DCOG-ALL treated *ETV6-RUNX1* cases after induction (TP1; n=65) and first consolidation course (TP2; n=66). The percentage of cases with high ($\geq 10^{-3}$), medium ($10^{-3} > \text{MRD} \geq 10^{-4}$), and undetectable MRD levels ($<10^{-4}$) is depicted per CNA.

Fisher's Exact test. **p≤0.01, *p≤0.05. del = deletion.

Supplementary Figure 6. The association between any CNA and MRD levels after induction therapy and the first consolidation course.

MRD levels of DCOG-ALL treated BCP-ALL cases after induction (TP1; n=183) and first consolidation course (TP2; n=183). The MRD levels of cases with any or no CNA are depicted for all subtypes, *BCR-ABL1*-like and non-*BCR-ABL1*-like B-other, high hyperdiploid, and *ETV6-RUNX1*.

Supplementary Figure 7. Prognostic value of CNAs in DCOG-ALL10 treated cases.

The association between CNAs and cumulative incidence of relapse (CIR) or event-free-survival (EFS) was examined. Patients were treated according to DCOG-ALL10 protocol. CIR was estimated using a competing risk model. Relapse and non-response were considered as event, and death as competing event. To test equality of the CIRs, the Gray's test was applied. Non-response, relapse, and death were considered as events for EFS. EFS rates were determined using Cox regression, and compared using the Wald test.

(**A**) CIR and EFS curves of cases without or with a *RB1* deletion. Curves contain either all risk groups, or the medium risk arm only.

(**B**) CIR and EFS curves of *BCR-ABL1*-like and non-*BCR-ABL1*-like B-other cases without or with *BTG1* deletions.

Supplementary Figure 8. Cellular drug response and CNAs

Leukemic cells were incubated for four days with an increasing concentration range of prednisolone (µg/ml), vincristine (µg/ml), L-asparaginase (IU/ml), daunorubicin (µg/ml), 6-mercaptopurine (µg/ml), and 6-thioguanine (µg/ml), after which cell viability was measured using an MTT assay. Association between indicated CNAs and *ex vivo* cytotoxicity was studied. The Mann-Whitney U test was applied to compare LC50-values. * $p < 0.05$, ** $p < 0.01$

(**A**) LC50 values of L-asparaginase (IU/ml) of primary leukemic cells without or with *IKZF1* deletion. Columns include all BCP-ALL subtypes (grey), BCR-ABL1-like/ B-other cells (blue), and high hyperdiploid cells (green). The red line represent the median LC50 value in the each group.

(**B**) LC50 values of prednisolone (µg/ml) of primary leukemic cells without an *IKZF1* or *PAX5* CNA, with *IKZF1* and *PAX5* CNA, a *PAX5* CNA without an *IKZF1* del, or *IKZF1* an del without a *PAX5* CNA. The red and black lines represent the median LC50 value in the each group.

(**C**) LC50 values of L-asparaginase (IU/ml) and 6-thiogunanine (µg/ml) of primary leukemic cells without or with *ETV6* deletion. Columns include all BCP-ALL subtypes (grey), BCR-ABL1-like/ B-other cells (blue), high hyperdiploid cells (green), and *ETV6-RUNX1*-positive cells (yellow). The red line represent the median LC50 value in the each group.

Supplementary Figure 9: The associations between the *IKZF1*^{plus}, long-term prognosis, MRD levels, and cellular drug resistance.

(**A**) Cumulative incidence of relapse and event-free survival curves are shown for patient enrolled in the DCOG-ALL10 protocol. The p-values of the curves of *IKZF1*^{plus}, *IKZF1*^{del} only, and *IKZF1*^{del} groups compared to the *IKZF1* wildtype group are reported. The dotted line (*IKZF1*^{del}) represents the total group of *IKZF1*^{plus} and *IKZF1*^{del} only cases (n=30). (**B**) MRD levels of DCOG-ALL10 treated BCP-ALL cases (all risk groups) after induction (TP1; n=183) and first consolidation course (TP2; n=183). The percentage of cases with high (≥10-3), medium (10 4 ≤ MRD < 10 3), and low MRD levels (<10-4) is depicted. (**C**) Leukemic cells were incubated for four days with a concentration range of prednisolone (µg/ml), vincristine (µg/ml), L-asparaginase (IU/ml), daunorubicin (µg/ml), 6-mercaptopurine (µg/ml), and 6 thioguanine (µg/ml), after which cell viability was measured using an MTT assay. LC50 values are

depicted. The red line represent the median LC50 value in the each group. * p < 0.05, ** p < 0.01; p-values against the IKZF1 wildtype group are reported.

Note: *IKZF1*^{plus}: presence of an *IKZF1*^{del} combined with loss of *PAX5* and/or *CDKN2A/B* (loss of PAR1 was not observed in this group). *IKZF1*^{del} only: cases with an *IKZF1* deletion that do not fulfill the *IKZF1*^{plus} criteria; *IKZF1*^{del}: presence of an *IKZF1* deletion involving both *IKZF1*^{plus} and *IKZF1*^{del} only cases.

Supplementary Figure 10: Integrated cytogenetic and genomic risk stratification in the DCOG-ALL10 cohort.

Cases were classified based on the risk stratification described by Moorman *et* al[1], which incorporates cytogenetic and CNA data. (**A**) Cumulative incidence of relapse and event-free survival curves of the UK good risk score and UK poor risk score are shown. (**B**) MRD levels of DCOG-ALL10 treated BCP-ALL cases after induction (TP1; n=183) and first consolidation course (TP2; n=183). The percentage of cases with high ($\geq 10^{-3}$), medium ($10^{-4} \leq$ MRD $< 10^{-3}$), and undetectable MRD levels ($<10^{-4}$) is depicted. (**C**) Leukemic cells were incubated for four days with a concentration range of prednisolone (µg/ml), vincristine (µg/ml), L-asparaginase (IU/ml), daunorubicin (µg/ml), 6-mercaptopurine (µg/ml), and 6-thioguanine (µg/ml), after which cell viability was measured using an MTT assay. LC50 values are depicted. The red line represents the median LC50 value in each group. * p < 0.05, ** p < 0.01

Supplementary Figure 1. Overview of cohort screened for CNAs, cellular drug resistance, and clinical outcome parameters.

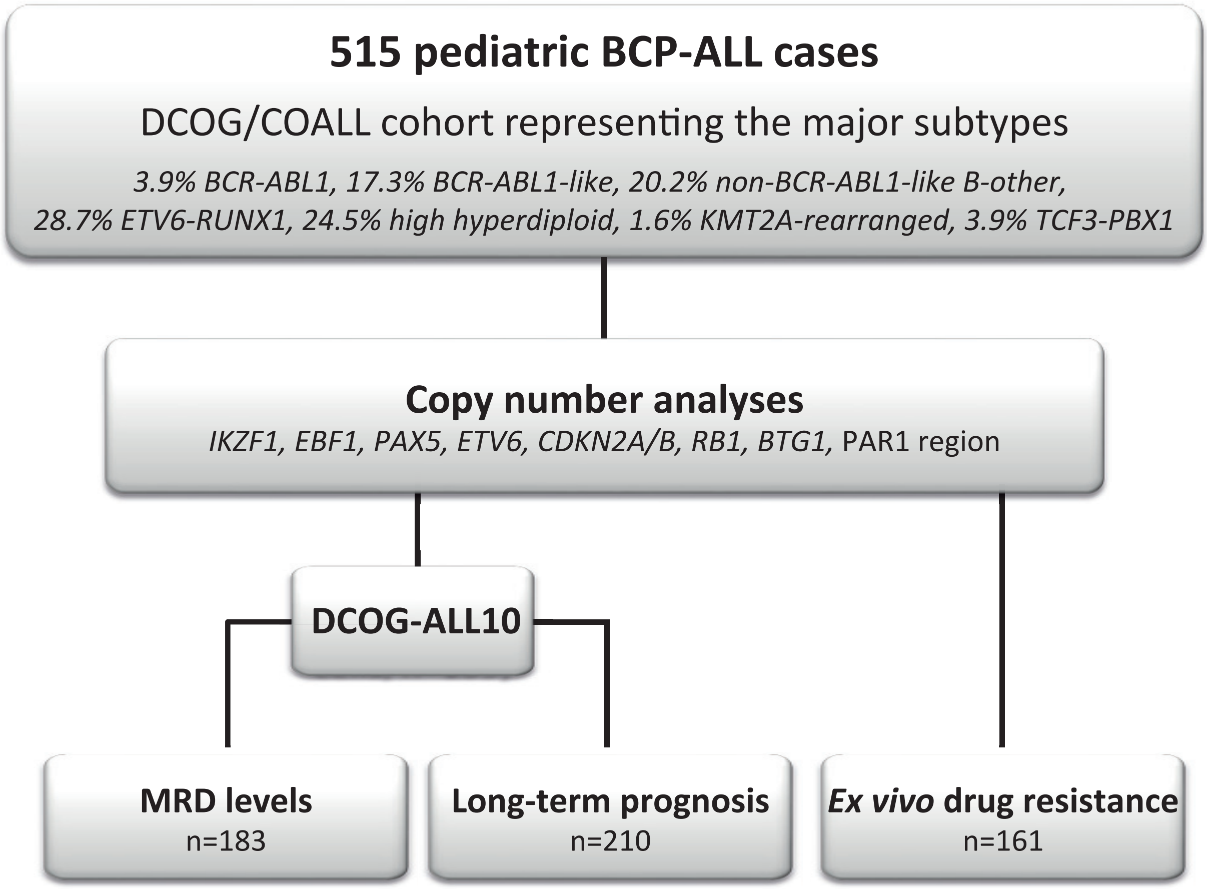

Supplementary Figure 2. CNA landscape of B-cell development genes in the different subtypes of pediatric BCP ALL.

A

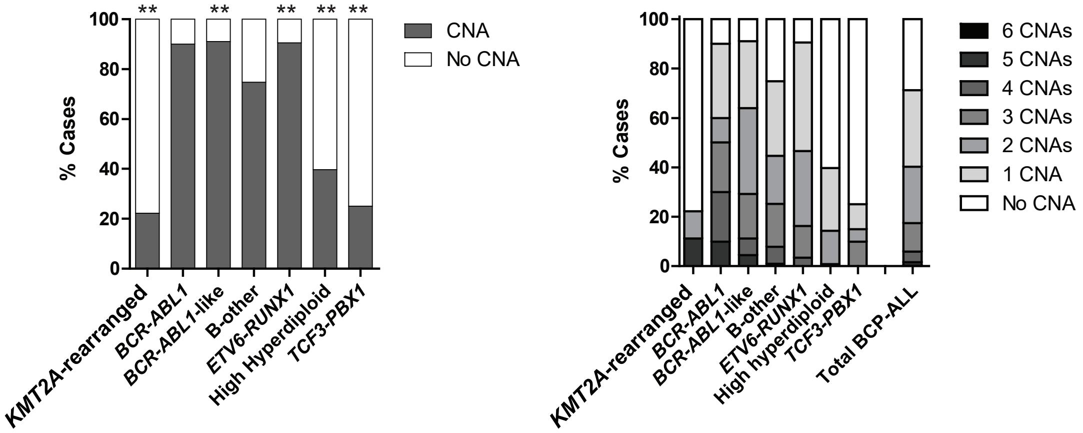

B

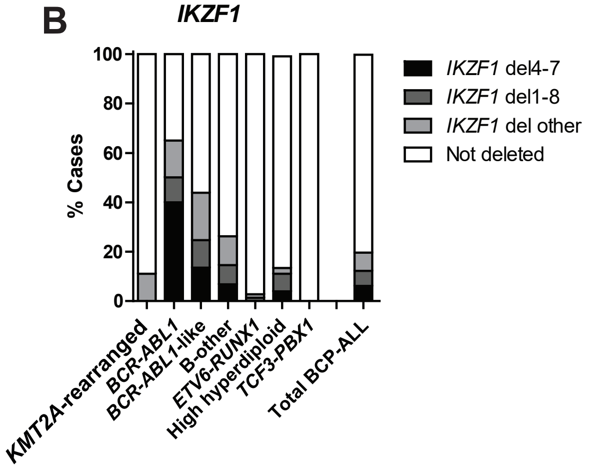

Supplementary Figure 3. The association between CNAs and MRD levels after induction therapy and the first consolidation course in *BRC-ABL1*-like and B-other patients.

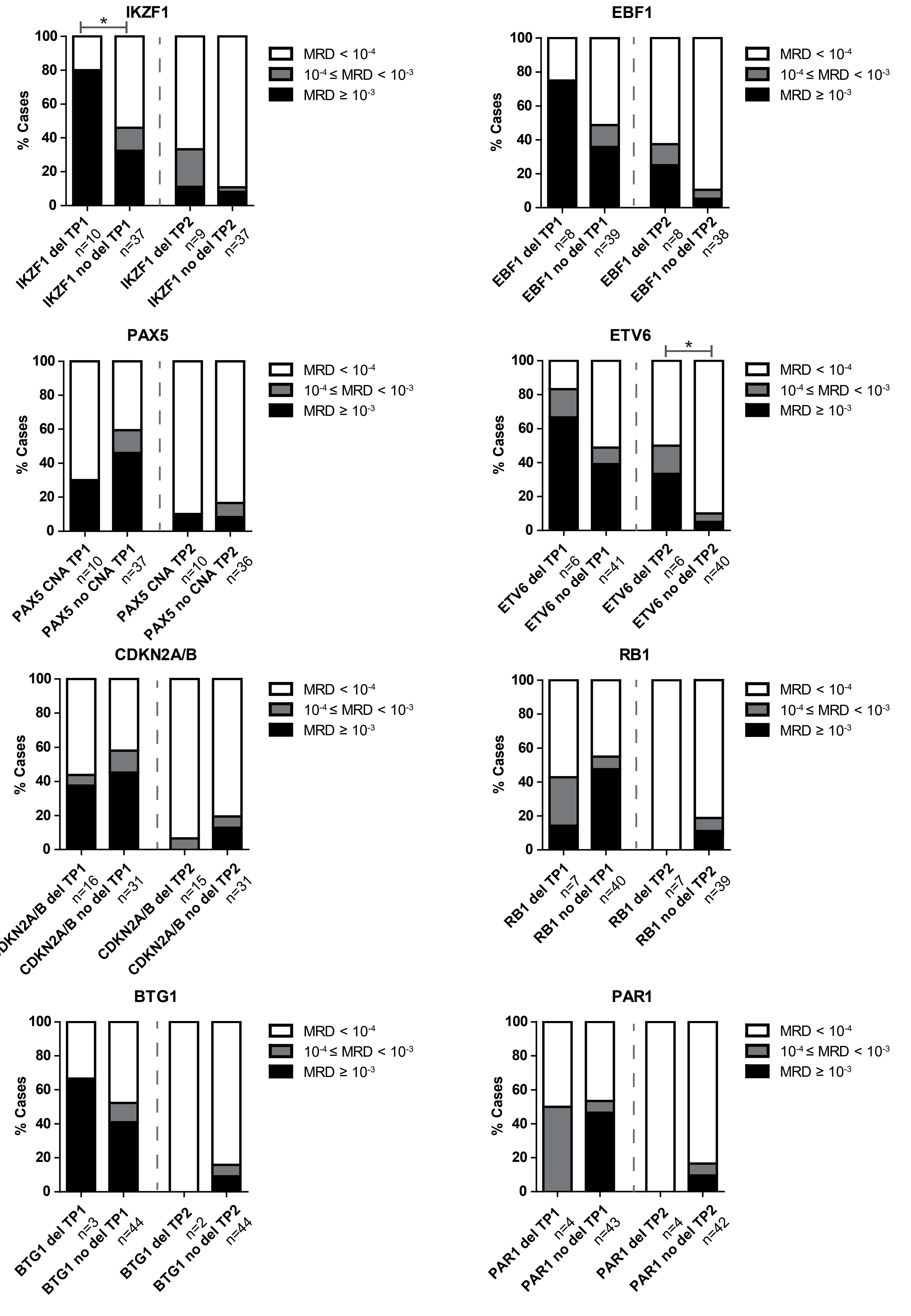

Supplementary Figure 4. The association between CNAs and MRD levels after induction therapy and the first consolidation course in high hyperdiploid patients.

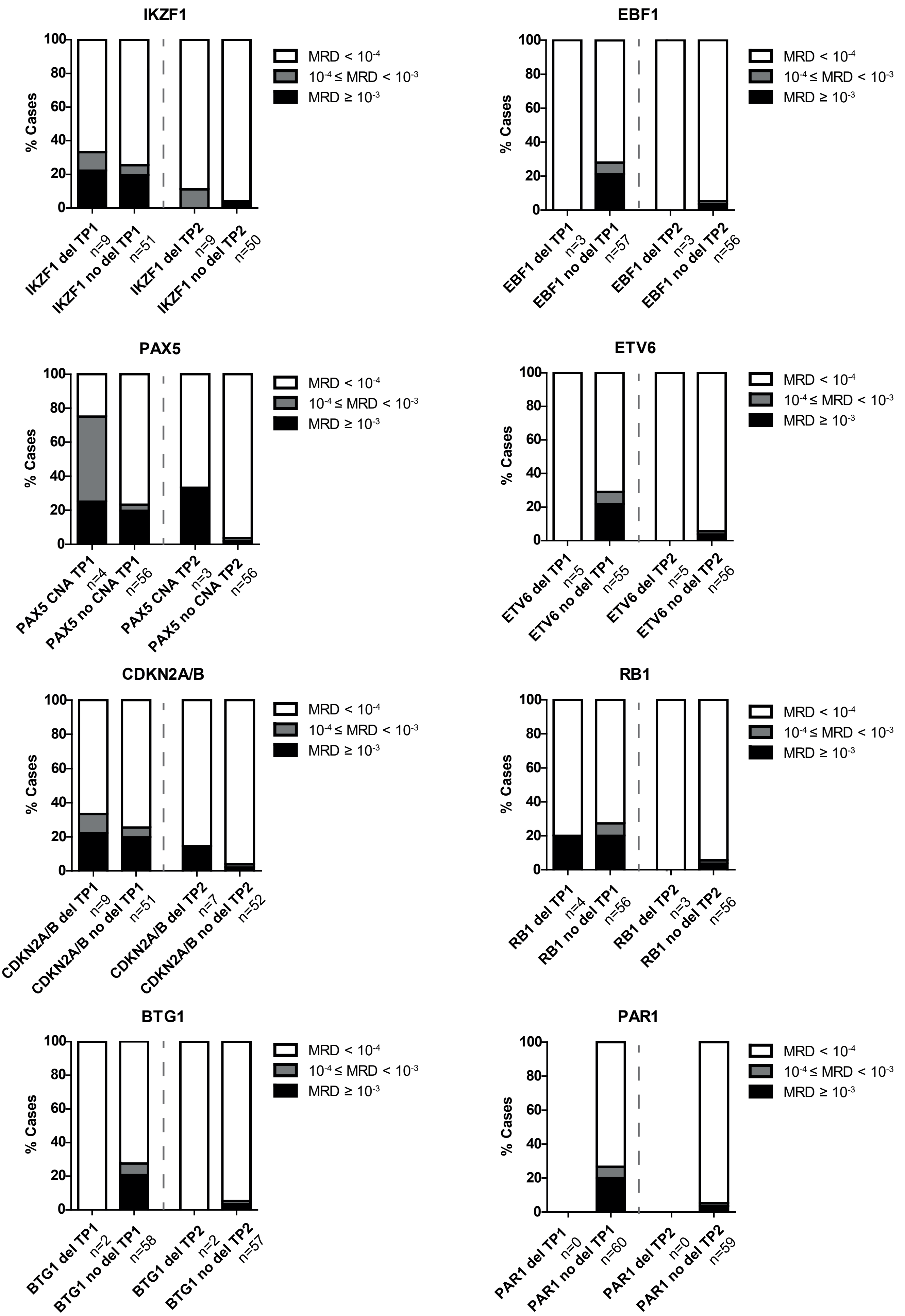

Supplementary Figure 5. The association between CNAs and MRD levels after induction therapy and the first consolidation course in *ETV6-RUNX1* patients.

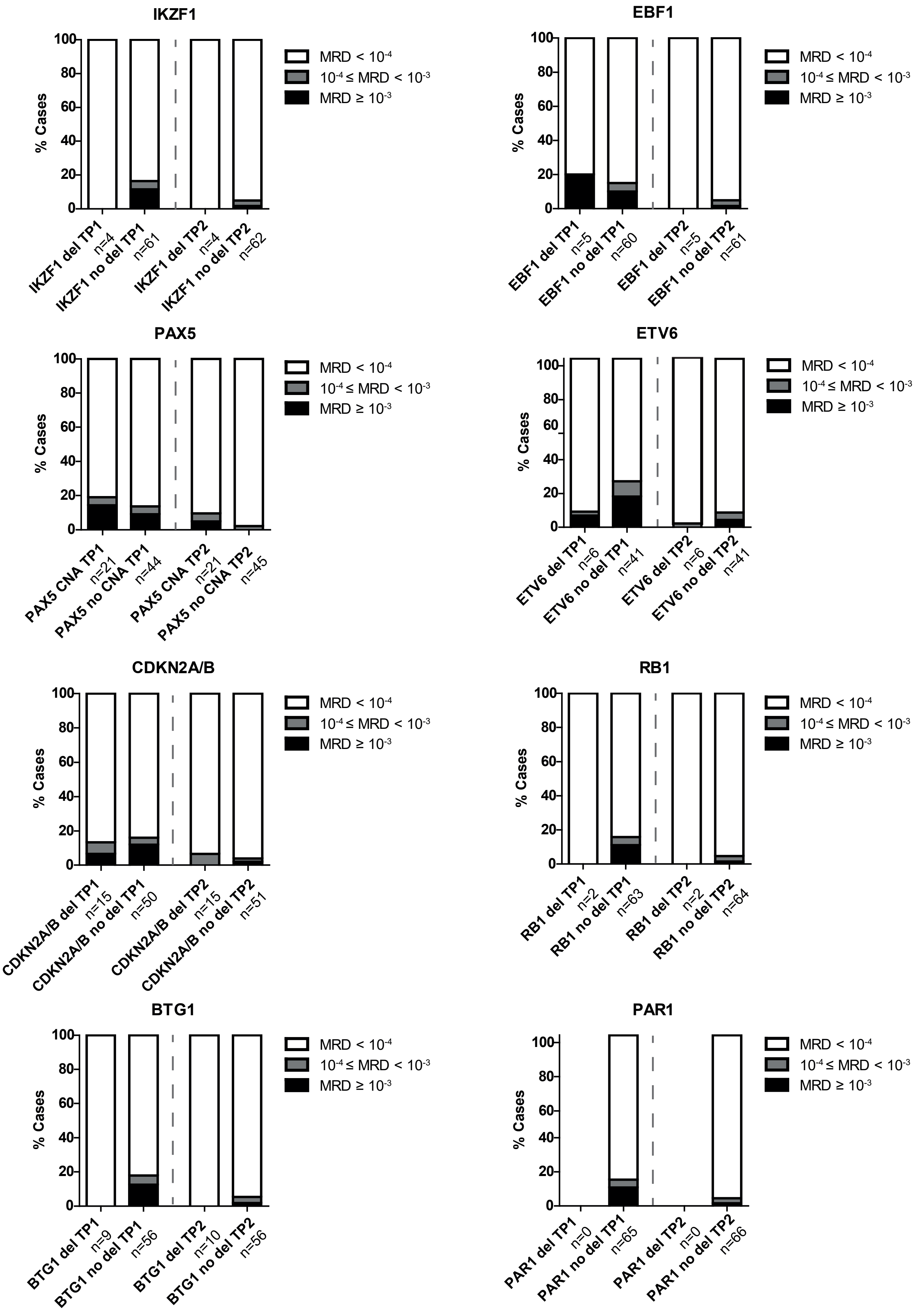

Supplementary Figure 6. The association between any CNA and MRD levels after induction therapy and the first consolidation course.

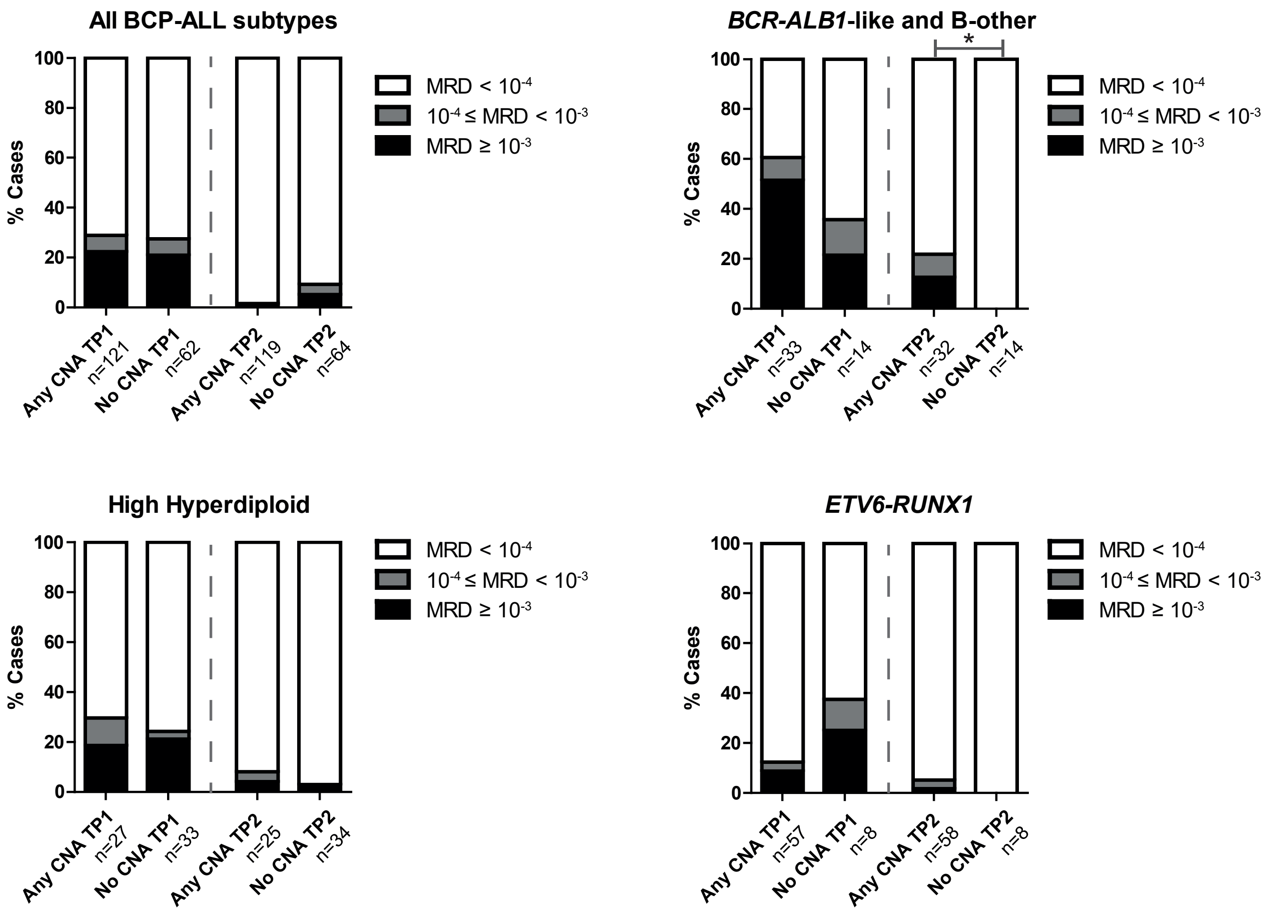

Supplementary Figure 7. Prognostic value of CNAs in DCOG-ALL10 treated cases.

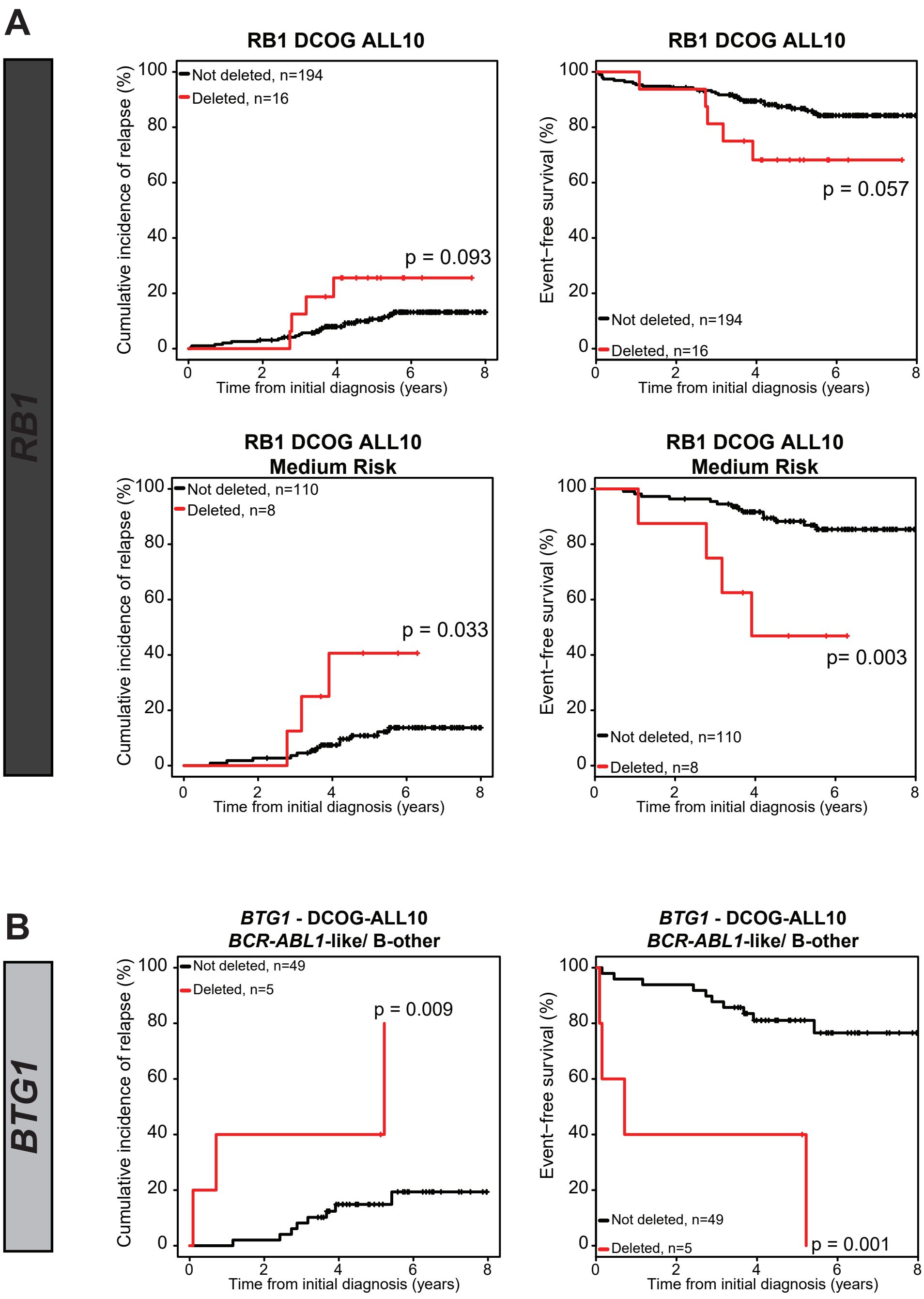

Supplementary Figure 8. The association between CNAs and the *ex vivo* cellular drug response.

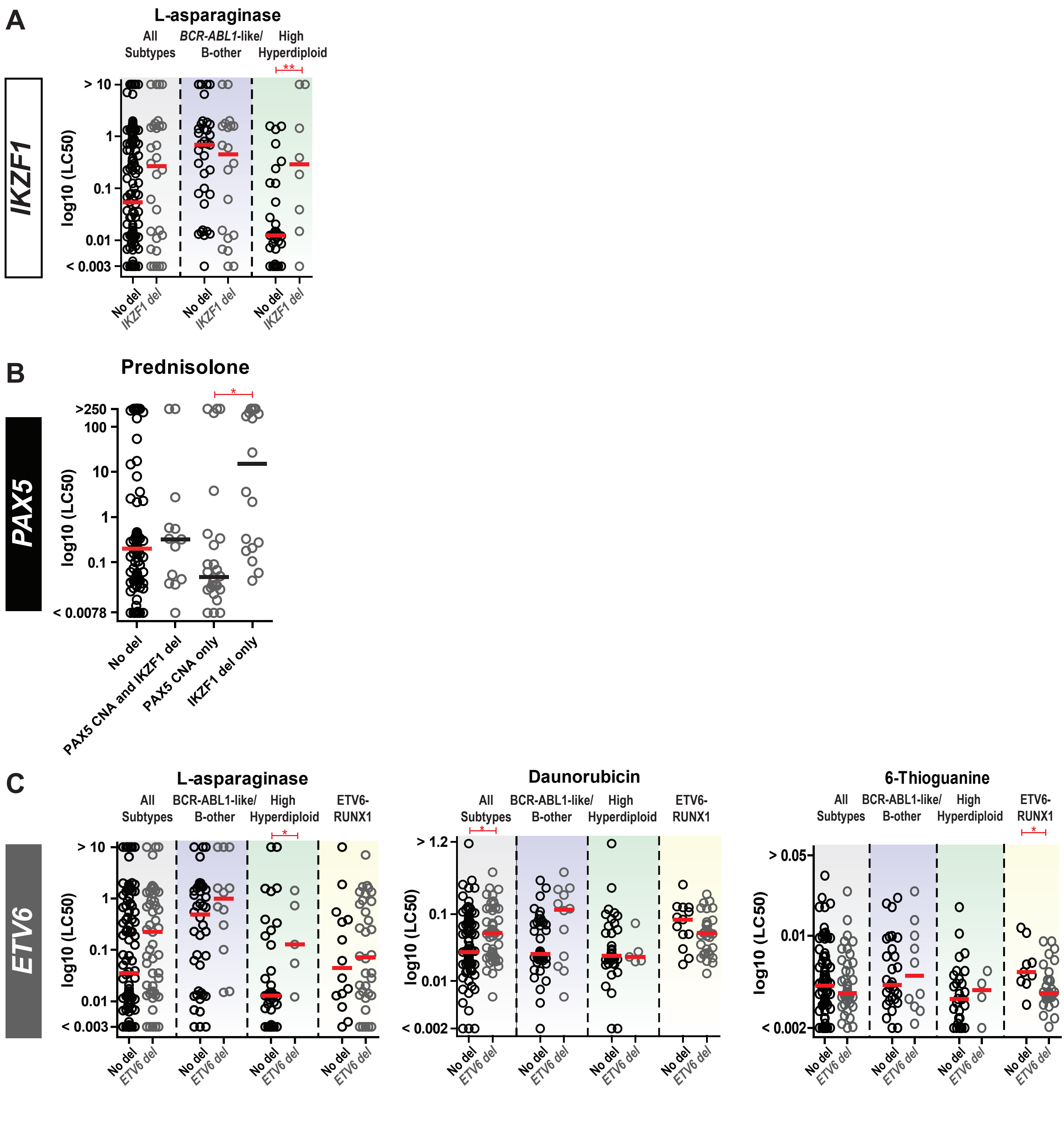

Supplementary Figure 9. *IKZF1*plus classification: prognostic relevance and cellular drug resistance.

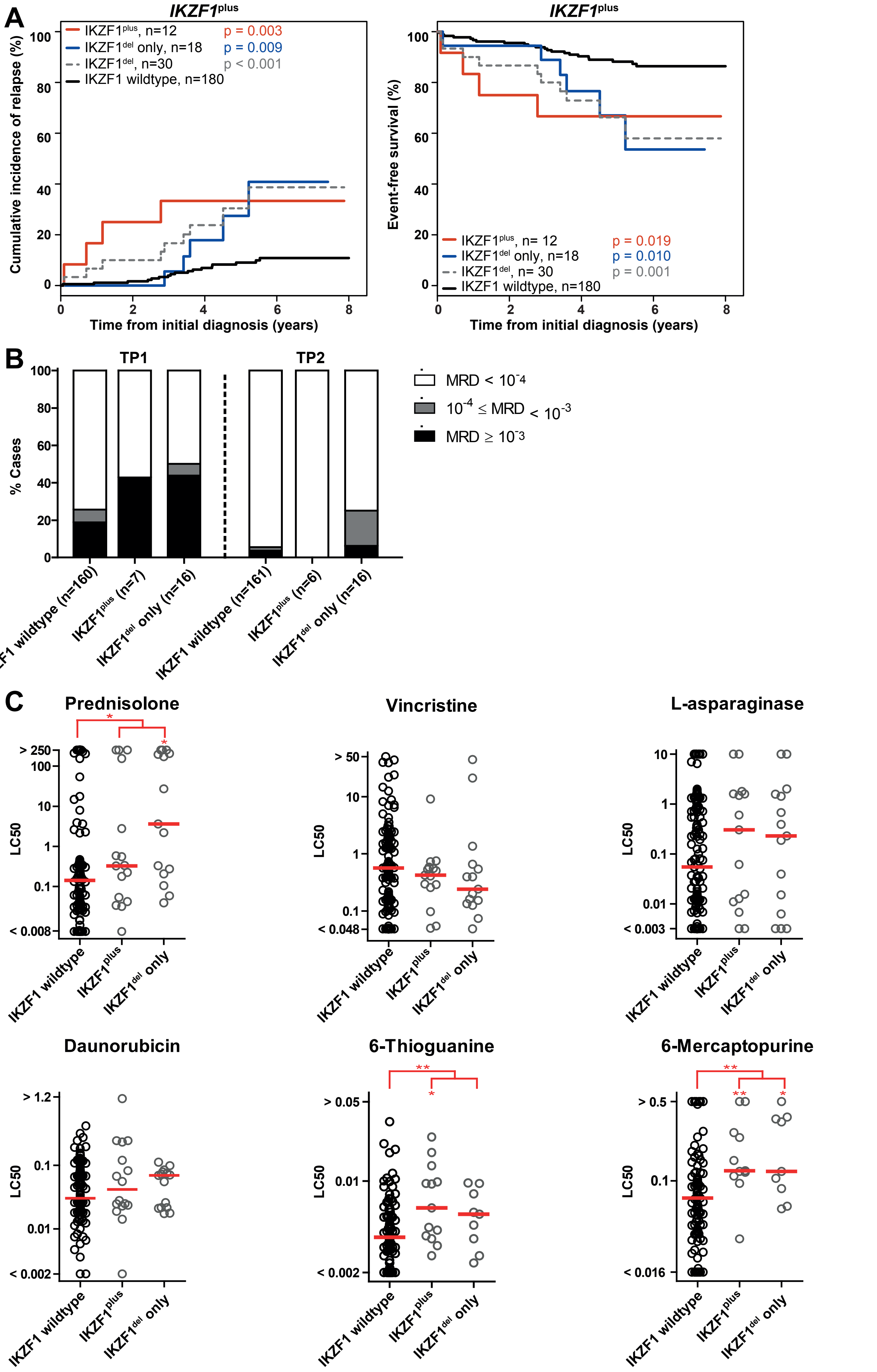

Supplementary Figure 10. Integrated cytogenetic and genomic risk stratification in the DCOG-ALL10 cohort.

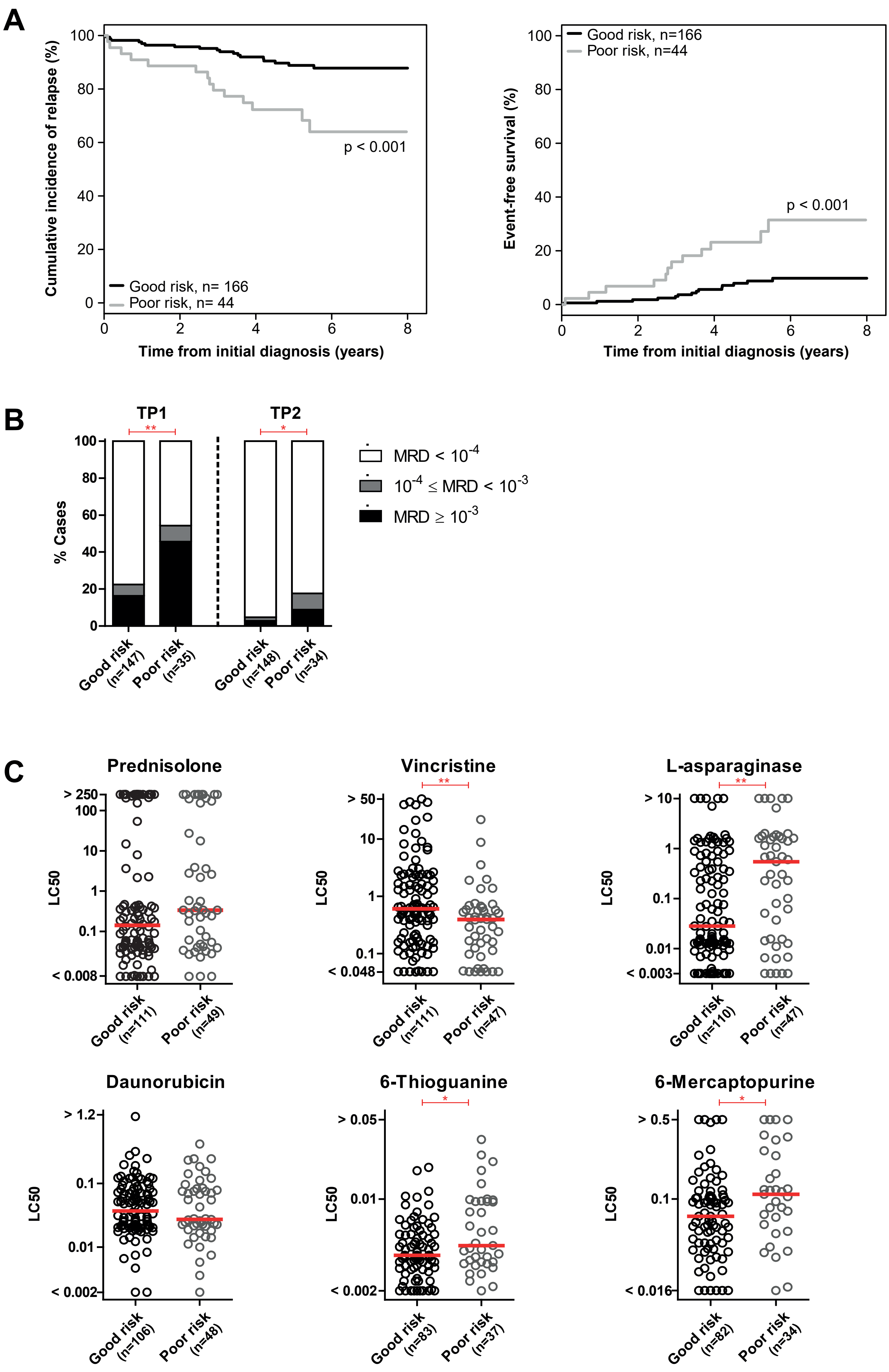

Supplement: Supplementary file 1 — Supplementary Information [file 41598_2019_41078_MOESM1_ESM.pdf]
